# Synthesis, Physicochemical Characterization, and Antibacterial Performance of Silver—Lactoferrin Complexes

**DOI:** 10.3390/ijms23137112

**Published:** 2022-06-26

**Authors:** Oleksandra Pryshchepa, Paweł Pomastowski, Katarzyna Rafińska, Adrian Gołębiowski, Agnieszka Rogowska, Maciej Monedeiro-Milanowski, Gulyaim Sagandykova, Bernhard Michalke, Philippe Schmitt-Kopplin, Michał Gloc, Renata Dobrucka, Krzysztof Kurzydłowski, Bogusław Buszewski

**Affiliations:** 1 Centre for Modern Interdisciplinary Technologies, Nicolaus Copernicus University in Torun, 87-100 Torun, Poland; p.pomastowski@umk.pl (P.P.); adrian.golebiowski@doktorant.umk.pl (A.G.); aga4356@wp.pl (A.R.); milanowski.maciej@gmail.com (M.M.-M.); sagandykova.gulyaim1@gmail.com (G.S.); b.busz@umk.pl (B.B.); 2 Department of Environmental Chemistry and Bioanalytics, Faculty of Chemistry, Nicolaus Copernicus University in Torun, 87-100 Torun, Poland; katraf@umk.pl; 3 Research Unit Analytical BioGeoChemistry, Helmholtz Zentrum Muenchen, 85764 Neuherberg, Germany; bernhard.michalke@helmholtz-muenchen.de (B.M.); schmitt-kopplin@helmholtz-muenchen.de (P.S.-K.); 4 Chair of Analytical Food Chemistry, Technische Universität München, 85354 Freising, Germany; 5 Faculty of Materials Science and Engineering, Warsaw University of Technology, 02-507 Warsaw, Poland; michal.gloc.wim@pw.edu.pl (M.G.); renata.dobrucka@pw.edu.pl (R.D.); 6 Department of Industrial Products and Packaging Quality, Institute of Quality Science, Poznań University of Economics and Business, 61-875 Poznań, Poland; 7 Faculty of Mechanical Engineering, Białystok University of Technology, 15-351 Białystok, Poland; k.kurzydlowski@pb.edu.pl

**Keywords:** sorption isotherm study, metalloproteins, silver nanoparticles, silver–lactoferrin nanocomplex, antibacterial properties, cytotoxicity

## Abstract

Antibiotic-resistant bacteria pose one of the major threats to human health worldwide. The issue is fundamental in the case of chronic wound treatment. One of the latest trends to overcome the problem is the search for new antibacterial agents based on silver. Thus, the aim of this research was to synthesize the silver-lactoferrin complex as a new generation of substances for the treatment of infected wounds. Moreover, one of the tasks was to investigate the formation mechanisms of the respective complexes and the influence of different synthesis conditions on the features of final product. The batch-sorption study was performed by applying the Langmuir and Freundlich isotherm models for the process description. Characterization of the complexes was carried out by spectroscopy, spectrometry, and separation techniques, as well as with electron microscopy. Additionally, the biological properties of the complex were evaluated, i.e., the antibacterial activity against selected bacteria and the impact on L929 cell-line viability. The results indicate the formation of a heterogeneous silver–lactoferrin complex that comprises silver nanoparticles. The complex has higher antibacterial strength than both native bovine lactoferrin and Ag^+^, while being comparable to silver toxicity.

## 1. Introduction

Pathogenic bacteria annually cause a significant number of human infections and deaths [1], where chronic wounds infections affect 1–2% of the population. They belong to the type of diseases that significantly decrease life quality. Patients with chronic wound infections complain about pain and limitation in physical activity. Moreover, such medical conditions are connected with the constant need for professional medical care, which negatively affects both the daily schedule and personal finances [2]. Wound-infection treatment includes the utilization of antibiotics, and the chronic character of the process leads to appearance of multidrug-resistant microorganism strains. Over 70% of infections are caused by bacterial strains resistant to one or more commonly used antibiotics. Thus, the development of metal-based preparations with antimicrobial properties, especially in the form of nanoparticles (NPs), have been of high interest recently among researchers [3]. 

Metal NPs such as silver [4] or zinc oxide [5] mostly reveal higher antimicrobial activity than respective ions. In the case of silver-nanoparticle (AgNPs) utilization, the advantages also account for the fact that to date no pathogenic bacteria that have total resistance against silver have been identified. However, metal NPs exhibit a wide spectrum of toxic effects on organisms, which restricts their utilization [4]. Still, in the case of palliative wound healing, any strategies that may reduce patients’ suffering are acceptable [6]. Moreover, NPs can be utilized as drug carriers. The combined effect of NPs with other antibacterial agents has shown to have a synergistic effect against pathogenic bacteria [7]. For instance, Rogowska et al. revealed that AgNPs functionalized with ampicillin had lower minimum inhibitory concentration (MIC) in comparison to both free NPs and ampicilline [8]. The approach enables the reduction of the required amount of utilized NPs to achieve positive results. Thus, the toxic effects caused by treatment also can be reduced. The reduced toxicity was also reported for biologically synthesized NPs, e.g., produced with utilization of probiotic bacteria, proteins, plant extracts, etc. [4]. 

The interactions of Ag^+^ with proteins have been investigated previously [9,10,11,12]. The Ag^+^ according to the Lewis theory belongs to soft acids and can interact with the soft basis, which includes, among others, sulfur-containing groups. This knowledge can be utilized for the prediction of metal binding to the protein-binding sites. The interactions of soft-acid metals with soft bases usually have covalent character [12]. It was shown that Ag^+^ can rapidly split disulfide bridges and form bonds with proteins through cysteines. The same was also observed only for Hg^+^ [9]. However, it is noteworthy to mention that in Pomastowski et al.’s work, the molecular modeling of the Ag^+^ interaction with bovine lactoferrin (bLTF) was performed [10]. The study revealed the significant impact of glutamic and aspartic acids in the Ag^+^ binding by protein. Still, earlier studies showed that Ag^+^ forms stronger bonds with N-containing functional groups rather than with O-containing ones [9]. Moreover, the distinctive feature of silver compounds is that they usually are photosensitive, i.e., they undergo reduction with the formation of metallic silver. The most popular Ag^+^ salt silver nitrate is not photosensitive, but the presence of organic traces promotes photoreduction [13]. Thus, interaction of Ag^+^ with proteins usually leads to the formation of metallic AgNPs [4,10,11].

In turn, bLTF is a non-heme iron-binding metalloprotein from the transferrin family. It is assumed that the main function of all transferrins is Fe^3+^-ion transport and regulation. However, lactoferrin (LTF) is also known for its role in host immune response during infections and inflammation [14]. Moreover, LTF also possesses antimicrobial [15], antiviral [16], antifungal [17], immunoregulatory and anti-inflammatory properties [18]. LTF has shown to be involved in cell proliferation and migration, among others, and topical administration of bLTF enables enhancement of the wound-closure process [19]. Several mechanisms of antibacterial properties have been reported for LTF. The ability to sequester Fe^3+^ with high efficacy affects growth of some Gram-positive and Gram-negative bacteria [19]. However, the activity is dependent on the iron saturation level and is abrogated in the case of holo-LTF. Moreover, it was revealed that bLTF can compete with lipopolysaccharide Ca^2+^-binding sites on bacterial membranes, which are involved in the outer cell-membrane stabilization. The sialic acid of bLTF glycans plays a role in Ca^2+^ chelating, thus indicating the importance of the protein glycosylation. Additionally, the presence of high-mannose structures on bLTF may nonspecifically prevent pathogen interaction with eukaryotic cells [20]. The antibacterial properties of LTF are also attributed to its highly cationic character. The beginning of the N-lobe contains a sequence with high content of arginines and lysines, which forms a so-called “cationic loop”. The cleavage of the LTF by pepsin can release the respective peptide called lactoferricin. The binding of either LTF or lactoferricin to the bacteria membrane disturbs its function [19]. In connection with the presented information, the use of bLTF for the production of antibacterial protein-based metallocomposites seems attractive.

Silver nitrate is the most popular precursor utilized in the synthesis of nanocomposites with antibacterial properties. However, silver ions (Ag^+^) of this precursor exist only in acidic conditions, while the increased pH above 6 causes the formation of insoluble silver oxide (Ag_2_O) [21]. In turn, it was shown that the interaction of metals with proteins occurs more effectively at higher pH [9,22]. The respective nanocomposites are also more stable in basic pH. In the previous work of our group [10], the investigation on the formation of Ag-LTF nanocomposite was performed with the utilization of the so-called “Tollens’ reagent”, i.e., silver in the form of a complex with ammonia [Ag(NH_3_)_2_]^+^. The utilization of silver complexation with ammonia enables the solubilization of silver in basic conditions, but significantly complicates the chemistry of the process. Therefore, it is interesting to investigate how bLTF will interact with Ag^+^ in acidic conditions. Moreover, the impact of different Ag^+^ concentrations on the features of the respective Ag-bLTF complexes should be studied. 

Thus, the aim of the study was to synthesize Ag-bLTF complexes and evaluate their biological activity, in particular antibacterial performance. Moreover, one of the tasks was to investigate the formation mechanisms of the respective complexes and the influence of different synthesis conditions on the features of final product. The work presents the results of the batch-sorption study performed by applying the Langmuir and Freundlich isotherm models for the process description. Meanwhile, characterization of the complexes was carried out by spectroscopy (ATR-FTIR, Raman, Fluorescence), spectrometry (MALDI-TOF-MS, ICP-MS), and separation (SDS-PAGE, CE-ICP-MS) techniques, as well as with electron microscopy (STEM-EDX, HTREM-SAED). The results enabled us to describe the process of Ag^+^ immobilization onto protein and to predict the possible mechanism of metal–protein interactions. The possible negative effects of the Ag-bLTF on living organisms were also evaluated by metal desorption analysis and cytotoxicity against the L929 cell line. 

## 2. Results and Discussion

### 2.1. Batch-Sorption Study

**Figure 1:**
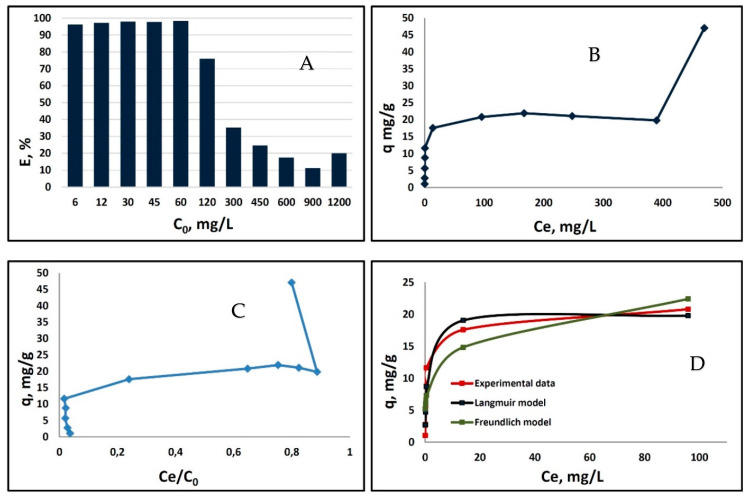
Batch isotherm study for Ag^+^ sorption onto bLTF: (**A**) isotherm of Ag^+^ adsorption onto bLTF for entire concentration range; (**B**) bar chart of a sorption efficiency of metal onto bLTF depending on the initial concentration of Ag^+^; (**C**) modified BET isotherm, which presents adsorption as a function of a ratio C_e_/C_0_; (**D**) the fit of first sorption step to Langmuir and Freundlich models.

The isotherm study enabled us to evaluate the maximum sorption capacity and other sorption parameters of bLTF in utilized conditions. The isotherm of Ag^+^ sorption onto bLTF and bar chart of a sorption efficiency for the entire range of utilized metal concentrations are shown in Figure 1A,B, respectively. The isotherm has a classical shape distinctive for multilayer adsorption, and according to IUPAC classification can be assigned to isotherm type II [23].

In turn, Figure 1C presents the modified BET (Brunauer–Emmet–Teller) isotherm proposed by M. Sprynskyy et al. [24]. Previously, the use of such an approach was successfully applied for the description of multilayer metal sorption onto proteins [11,25]. In general, in both presented works the real character of the sorption was not possible to specify only by the shape of the classical isotherm. Instead, the modified BET isotherm enabled the clear outlining of the concentration ranges required for the formation of each subsequent layer. In the present study, the approach did not provide much additional information. Still, it was possible to distinguish two adsorption layers, where the first one was observed for the initial Ag^+^ concentrations up to 900 mg/L. Meanwhile, the beginning of the second adsorption layer started with utilization of initial Ag^+^ concentration of 1200 mg/L. The kinetic studies performed by P. Pomastowski et al. also confirmed the complex character of the process. Their investigation indicated that three stages of the sorption process can be distinguished. The first initial rapid-sorption step is related to the surface sorption, which is followed by gradual sorption with rate-limiting intraparticle diffusion. Finally, the process ends up with the equilibrium stage. Interestingly, in the P. Pomastowski et al. studies silver solution with concentration of 50 mg/L was utilized, and respective adsorption reached 73.6% and 8.1 mg/g [10]. Meanwhile, in the present study for the initial concentration of 45 and 60 mg/L, the adsorption efficiency was ≈98% in both cases and respective adsorption accounted for 8.78 and 11.62 mg/g, respectively. It can be concluded that silver in the form of amino complex is less available for the interaction with protein. Instead, acidic conditions have a low impact on the sorption efficiency of Ag^+^ by bLTF. 

Further, the Langmuir and Freundlich isotherm models were applied. The respective models are simple, and thus are often used for the description of the sorption processes. Previously, the approach was successfully utilized to derive sorption parameters for Ag^+^ and Zn^2+^ binding to caseins, Ova-albumin, and β-lactoalbumin [11,25,26]. However, it is noteworthy to mention that the Langmuir and Freundlich models can only be applied to isotherm type I [23]. Thus, we utilized it to our investigation, but only for the first step of the obtained isotherm (Figure 1D). The higher value of the correlation coefficient R^2^ was obtained for the Langmuir model (significance level *p* > 0.05), indicating its better fit to experimentally derived data. The Langmuir model assumptions are the monolayer character of the adsorption and homogeneous surface in terms of binding-site energies [23]. Conversely, the Freundlich model assumes the heterogeneous nonideal character of the adsorption process and is not restricted to monolayer formation [27]. For the respective model, the correlation coefficient R^2^ was only 0.905, but the significance level *p* < 0.02.

Interestingly, the Ag^+^ adsorption onto bLTF was calculated as 47.06 ± 0.25 mg/g for the initial metal solution with concentration of ≈1200 mg/L, while the ICP-MS analysis of the respective complex (AgLTF1200) revealed that only 2.48 ± 0.46 mg/g (differing slightly from batch to batch) of silver remain after the washing steps. Additionally, even though the Ag^+^ binding process was performed in acidic conditions, it did not cause significant loss of iron. The Fe^3+^ content in the complex was 1.31 ± 0.13 mg/g, which is almost the same as in native bLTF (1.45 ± 0.06 mg/g, protein-to-metal ratio ≈1:2 [22]). The results show the surface character of Ag^+^ binding and high correlation of the estimated silver content in the complex with derived *q_m_* from the Langmuir model. Moreover, this fact confirms the fairness of the utilization of the above-mentioned model in describing metal–protein interactions. Considering the protein molecular weight (≈82 kDa by MALDI-TOF MS) the Ag:bLTF ratio in the complex was determined which is 1.78:1. Table 1 summarizes the calculated values for the utilized models (derived by CurveExpert Basic 2.2.3 software). In the previous study on the Zn^2+^ binding to proteins, namely Ova-albumin and β-lactoglobulin, the formation of ZnONPs in first case and the formation of homogeneous metal-protein complex was observed [25,26]. For the Zn^2+^/ZnO-Ova nanocomposite, the same as in present study, the relatively high Langmuir constant and low *q_m_* were calculated, e.g., 1.95 L/mg and 10.97 mg/g. In comparison, for the homogeneous Zn-β-lactoglobulin complex, the respective parameters were 0.01 L/mg and 104.40 mg/g. Thus, in the case of Ag-bLTF interactions, the formation of AgNPs can be predicted. Still, for the silver interactions with caseins, the sorption parameters were more similar to Zn-β-lactoglobulin (e.g., 0.02 L/mg and 77.44 mg/g). This fact may be connected to caseins’ complex structure, which appears in the solution in the form of highly organized micelles [11]. 

The changes of the free Gibbs energies Δ*G* were calculated based on the value of the distribution coefficient (*K_D_*). In general, the data indicate that Ag^+^ binding to bLTF has spontaneous character. Moreover, some interesting correlations can be observed. For instance, in cases where solutions of low Ag^+^ concentration (6–60 mg/L) were utilized, the calculated Δ*G* were almost similar and were in the range between −22.76 and −24.741 kJ/mol. The lowest value was when the Ag^+^ concentration of 6 mg/L was used, which may be connected to the formation of AgNPs, since the formation of a new surface is a thermodynamically unfavorable process [4]. Instead, for Ag^+^ concentrations of 15–60 mg/L, the differences in calculated Δ*G* are in the range of standard deviation, which is complementary to the better fit of the Langmuir model to the experimentally obtained adsorption isotherm. For higher Ag^+^ concentrations (120–900 mg/L), the calculated Δ*G* values gradually decrease from −17.58 to −9.67 kJ/mol, which may indicate that some of the Ag^+^ bonds to bLTF through weak electrostatic interactions. Instead, the breaking of the energy barrier and formation of a new adsorption layer resulted in a slight increase in Δ*G* to −11.34 kJ/mol, which was observed for Ag^+^ solution with a concentration of 1200 mg/L. 

### 2.2. Physicochemical Characterization of Synthesized Complexes

#### 2.2.1. Electron Microscopy

**Figure 2:**
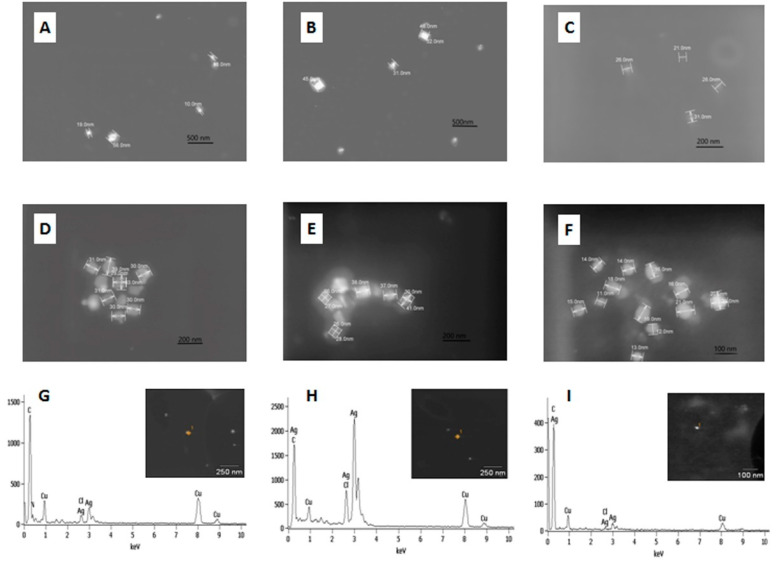
STEM images of AgLTF complexes with marked particle sizes: (**A**) AgLTF30; (**B**) AgLTF45; (**C**) AgLTF120; (**D**) AgLTF450; (**E**) AgLTF900; (**F**) and STEM-EDX spectra of samples: (**G**) AgLTF30, (**H**) AgLTF45, (**I**) AgLTF1200.

Silver ions tend to reduce in the presence of organic compounds with the formation of metallic nanoparticles. Thus, electron microscopy was utilized for the AgLTF complexes’ examination. Representative images of samples AgLTF30, AgLTF45, AgLTF120, AgLTF450, AgLTF600, and AgLTF1200 are shown in Figure 2. 

The images reveal the presence of mostly spherical silver nanoparticles in all studied samples of AgLTF. The size of the AgNPs fell within the range of 10–56 nm. It was noticed that samples obtained with lower Ag^+^ concentrations in the reaction mixture were characterized by the presence of less agglomerated, smaller, mostly spherical structures. Interestingly, for AgLTF1200 the presence of smaller nanoparticles with sizes <20 nm was observed. Moreover, the biggest NPs were noticed for the complexes AgLTF45-AgLTF900. 

The formation of NPs happens through a process comprised of several stages: nucleation, growth by Me^0^ incorporation, and aggregation [4]. Each of the stages is dependent on the time-resolved Me^0^ concentration and the amount of capping agent, which in consequence influences the size and size distribution of the formed NPs. Thus, by controlling the amount of free Ag^+^, the bLTF (acting both as the reducing and capping agent), and metal/protein ratio, the nanocomposite with the desired features can be obtained. Our studies show that with the increase in the silver in the reaction mixture at the stable bLTF concentration, the size of nanoparticles becomes bigger, up to an Ag^+^ concentration of 45 mg/L. This should indicate that a smaller amount of AgNP nuclei was formed but a higher rate of NP growth occurred. For reaction mixtures where Ag^+^ concentration was in the range between 45 and 900 mg/L, the NPs size was almost the same, but their amount was higher when higher metal concentrations were used. When the concentration of Ag^+^ reached 1200 mg/L, a higher amount of nuclei formed, but the Ag^0^ concentration may have been too low for NP growth. Thus, a large amount of AgNPs with smaller sizes were formed. As can be seen, the resulted features of the formed nanocomposites correlate with the isotherm-sorption studies. Further, EDX analysis was performed, which confirmed that the particles of interest contain a high concentration of silver. Similarly, as in previous works [1], the presence of copper was due to the use of a copper mesh for analysis and proteins for stabilization of the Ag nanoparticles. However, the EDX analysis also revealed the presence of chlorides in the sample. In the case of biogenic AgNPs, contamination with the chloride ion is one of the most common contaminations. AgCl is also photosensitive and its irradiation with daylight should also lead to the formation of metallic NPs in the end [28]. However, such NPs have the synthesis mechanism of other characters in comparison to the direct formation of AgNPs by reduction with functional groups of the protein. 

**Figure 3:**
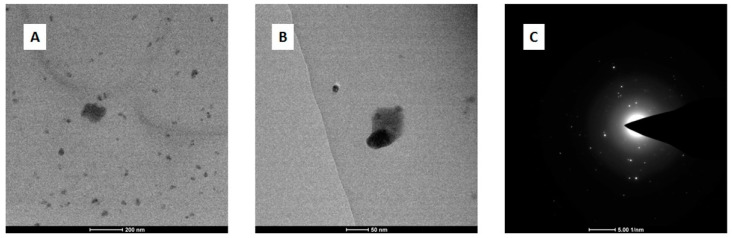
TEM images of AgbLTF450 at different magnifications (**A**,**B**) and (**C**) SAED pattern recorded for AgbLTF450.

The samples were also subjected to TEM-SAED analysis. Figure 3 shows TEM-images of the AgLTF450 and corresponding SAED pattern. From Figure 3A, it can be seen that the complex is comprised of a high amount of protein, which disturbs the SAED analysis. Thus, even though SAED reveals the crystalline structure of the formed AgNPs, it was not possible to determine the interplanar distances.

#### 2.2.2. Fourier Transform Infrared Spectroscopy (FTIR)

**Figure 4:**
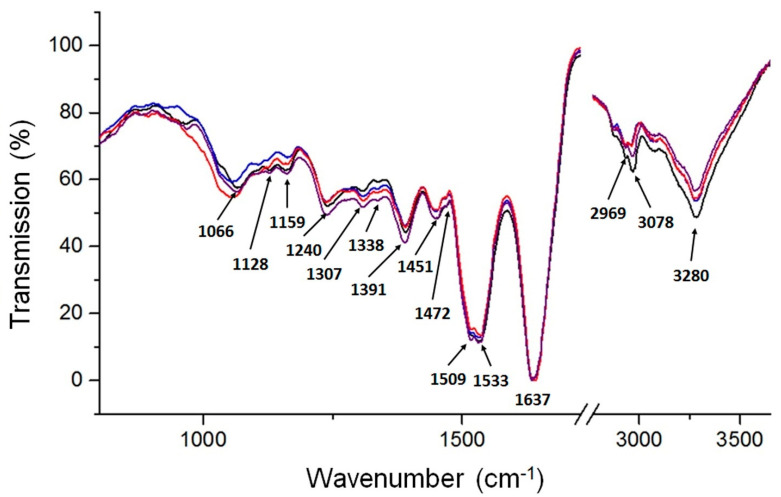
ATR-FTIR spectrum of native bLTF (black) and its complexes synthesized with silver solutions of 45 mg/L (blue), 450 mg/L (red), and 1200 mg/L (purple).

FTIR is a technique that allows the tracking of changes in absorbance of certain functional groups upon protein interactions [12]. Figure 4 presents the absorption spectra recorded for native bLTF and the complexes synthesized with utilization of Ag^+^ solutions with concentration of 45, 450, and 1200 mg/L. The spectrum of native bLTF reveals the pattern of absorption bands distinctive for proteins. The region 2800–3700 cm^−1^ presents the vibrations of hydrogen-containing groups. The wide band with a maximum at 3280 cm^−1^ corresponds to vibrations of O-H and N-H that take part in hydrogen bonds. The band at 3078 cm^−1^ comes from C-H in aromatic rings, while bands with maxima in the range from 2800–3000 cm^−1^ can be assigned to doublets of C-H asymmetric and symmetric vibrations of methyl and methylene groups [29]. The redshift and changes in the intensities of corresponding signals after bLTF interaction with Ag^+^ can be observed, which indicates the occurrence of structural modifications in the protein. Both the Ag^+^ and AgNPs’ incorporation to protein should induce such changes. For instance, the band at 2969 cm^−1^ of bLTF shifted to 2957, 2962, and 2964 cm^−1^ of AgLTF45, AgLTF450, and AgLTF1200 cm^−1^, respectively. The additional bands for respective groups also can be detected: δ_as_(CH_3_) at 1472 cm^−1^ and δ(CH_2_) at 1451 cm^−1^ [29,30]. Alhazmi et al. in their work based on the study performed on bovine serum albumin (BSA) also came to the conclusion that incorporation of any of the metal leads to the changes in protein structures [12].

The common protein bands of amide I and amide II for all samples were observed at 1637 cm^−1^ and 1533 cm^−1^. It is noteworthy to mention that the position of amide I indicates the structural features of the protein, and in case of bLTF shows the high content of the β-sheet in protein [30,31,32]. However, more accurate information about protein’s secondary structure can be deduced from amide III modes [30,32]. The spectrum for native bLTF reveals bands of amide III at 1338 cm^−1^, 1307 cm^−1^ which come from α-helix structures. Instead, more strong absorption at 1240 cm^−1^ of the β-sheet was observed. The corresponding bands did not shift after Ag^+^ binding to bLTF, but the slight broadening of the band at 1240 cm^−1^ was detected. Moreover, the band at 1276 cm^−1^ (random coils) for AgLTF450 became visible. Such band shifts, in combination with the alterations in band intensities, also revealed the changes in the protein structure [12,30]. Additionally, after Ag^+^ binding, the second maxima at 1509 cm^−1^ appeared in all studied complexes. The corresponding band may come from vibrations of tyrosine and tryptophane residues [30]. Moreover, the relative band intensities of ν_s_(COO^−^) of glutamic and aspartic acids (intense signal at 1391 cm^−1^) were also changed after bLTF interaction with Ag^+^. Interestingly, no shifts of respective bands were observed, while literature data indicate that they can shift significantly (+60/−90 cm^−1^) [29] upon metal chelation. Still, in Pomastowski et al. [10], the molecular dynamics and quantum mechanics calculations of Ag^+^ interaction with bLTF were performed, which indicate that aspartic and glutamic acids of bLTF were the dominant residues for Ag^+^ binding. Furthermore, the ν(C-O) vibrations of serine, threonine, glutamic, and aspartic acids in bLTF are presented by bands at 1159, 1128, and 1066 cm^−1^ [30]. In case of AgLTF45, the significant shifts and signal split were observed. The bands at 1163, 1124, 1107, 1100, and 1055 cm^−1^ were detected. On the spectrum of AgLTF450, the corresponding region was also distorted. The bands at 1155, 1148, 1125, 1105, and 1053 cm^−1^ were observed. In the case of AgLTF1200, the band with the maximum at 1065 cm^−1^ broadened significantly upon Ag^+^ binding. The respective band may overlay other signals in the studied absorption region. It should be noted that the band at 1105/1107 cm^−1^ can come from ν(CN) vibrations of histidine, indicating the influence of Ag^+^ on the respective amino acid [30]. Summing up, it can be assumed that all of the abovementioned amino acids should somehow be involved in the binding process. The bands presented in the region between 1000–900 cm^−1^ may come from ν(CO) or ν(CC) of serine. However, bLTF is a glycoprotein, the glycan content of which ranges from 6.7 to 11.2% of the total molecular weight [20]. Thus, the bands below 1000 cm^−1^ can also be assigned to ν(C-O), ν_st_(C-O), ν_st_(C-C) of glycans [33], while below 900 cm^−1^ may correspond to *ν*CO+*δ*CCH+*ν*_asy_ of the pyranose ring and *ν*CC+*δ*CCH+*δ*CHβ-pyranose of glycans. Therefore, the changes that occur in the corresponding region may be due to either interaction with side chains of respective amino acids or with components of protein glycans. It is worth mentioning that the main disadvantage of spectroscopy in the infrared range is that bands corresponding to several functional groups can appear in the same region. Hence, it makes it difficult to distinguish which functional group has the most significant impact on metal bonding by protein.

#### 2.2.3. Raman Spectroscopy

**Figure 5:**
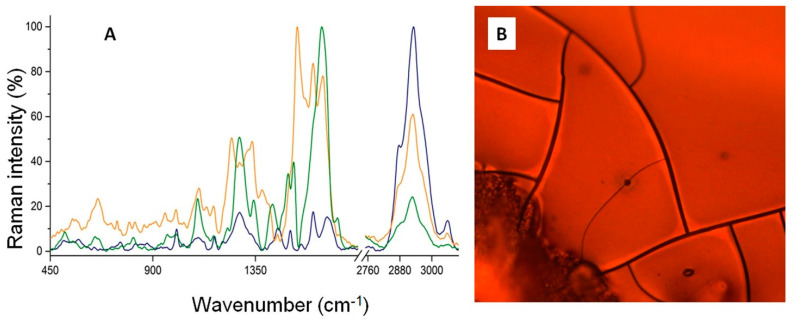
(**A**) Raman spectra of native bLTF (blue), AgLTF45 (green), and AgLTF450 (orange), (**B**) The picture of AgLTF1200 complex under microscope objective, where blue arrows show the places of protein degradation.

Some functional groups that are weak or inactive within FTIR can exhibit strong bands in Raman; thus, appropriate analysis was performed to complement the study. On the Raman spectrum (Figure 5) of native bLTF, the band at 3325 cm^−1^, the same as in FTIR, comes from O-H and N-H groups involved in hydrogen bonds [31], but has lower intensity. Similarly, C-H vibrational bands are at 2931 and 2873 cm^−1^. Instead, the band at 3055 cm^−1^ can be assigned to N-H str. vibrations. The analysis of the amide I and amide III bands’ positions in Raman can also provide a significant amount of information regarding the secondary structure of the protein. The amide I band was detected at 1664 cm^−1^, while the amide III region presented bands at 1280 cm^−1^ and 1329 cm^−1^ [34]. Likewise, in FTIR the position of the respective bands indicates the presence of all of the structures with prevalence of β-sheets [34,35]. The literature data reveal that the amide II band should rather have low-intensity Raman spectra [31,32]. However, the spectrum of native bLTF comprises the medium-intensity band at 1503 cm^−1^, which may be assigned to amide II vibrations. The relative band can also come from aromatic rings of indole or imidazole from tryptophan and histidine. Moreover, the imidazole of histidine may give the-low intensity band at 1391 cm^−1^, while indole ring modes of tryptophan are observed at 1550 cm^−1^. Other tryptophan bands can be detected at 874 and 755 cm^−1^ [34,35]. The band characteristic for aromatic amino acids, i.e., tryptophan, tyrosine, and phenylalanine, were also presented by a band at 1604 cm^−1^. Additionally, the tyrosine bands also appeared at 1206, 1171, and 835 cm^−1^. Instead, phenylalanine bands were detected at 1035, 1003, and 962 cm^−1^. The bands of C-H and C-N deformation stretching were observed at 1450 cm^−1^ and 1096 cm^−1^, respectively [35]. The region at 500–600 cm^−1^ is characteristic for stretching vibrations of different conformers of S-S bonds [34]. Finally, the bands that appear near ≈410 cm^−1^ come from Fe-O or Fe-NO complexes in iron-binding proteins [36].

After Ag^+^ binding to bLTF, the significant shifts and signal enhancement of bands in the fingerprint region can be observed on the spectra of AgLTF45 and AgLTF450 complexes. Instead, the Raman spectrum for the AgLTF1200 complex was not possible to obtain, as it undergoes burning under laser irradiation even with the lowest energy. The observed effect may be connected to the formation of Ag nanoparticles (AgNPs). A specific feature of the metallic NPs is the appearance of LSPR (localized surface-plasmon resonance) [4]. Surface plasmon of AgNPs can enhance the signals in Raman, which is the basis of a powerful technique, namely SERS (surface-enhanced Raman spectroscopy). The signal enhancement can be expected from the functional groups that are directly bonded to the NPs’ surface. Thus, according to obtained results, it may be deduced that a small number of incorporated NPs caused the increase in signal intensity of appropriate functional groups in AgLTF45 and AgLTF450 complexes. However, the LSPR is also the reason of the NPs’ catalytic activity, which should be a reason for the protein degradation in the AgLTF1200 complex within Raman. 

On the Raman spectrum of AgLTF45, the amide I band shifted significantly from 1664 to 1644 cm^−1^. Conversely, the most distinctive band for ring modes of all aromatic residues shifted only to 1602 cm^−1^, but increased significantly. Furthermore, the band with the maximum at 1532 cm^−1^ rose. The band is characterized with a wide base that occupies a range between ≈1480 and 1600 cm^−1^, and thus hides other bands visible on the spectrum of native bLTF. The corresponding band can be assigned for the indole ring modes that mainly come from C_2_=C_3_ stretching vibrations, and is affected by the torsion angle of about C3-Cβ and Cβ-Cα [37]. The obtained data, the same as in FTIR, may indicate Ag^+^ interaction with heteroatoms of peptide bonds. It is noteworthy to mention that Ag^+^ can coordinate both O and N, but forms stronger bonds with the latter [9]. Thus, Ag^+^ should preferably interact with nitrogen-containing groups. The spectrum recorded for AgLTF450 in the respective absorption range revealed even more drastic changes. The band at 1639 cm^−1^, which was assigned to the amide I vibrations, underwent an enormous increase, so it hid the band at ≈1600 cm^−1^. Moreover, on the spectrum the bands at 1513 and 1493 cm^−1^ can be distinguished, which may come from C=C of aromatic rings of tryptophan and histidine, respectively. The pioneer studies of Sidgwick in the field have revealed that a higher number of substituents in amines decreases the stability of its complexes with the metals. However, he also pointed out that pyridines and imidazole act rather as primary amines than as tertiary ones [9]. Thus, Ag^+^ should favor the interaction with histidines and tryptophans, which may be a reason for the mentioned changes on Ag-bLTF spectra. The band of C-H deformation vibrations at 1448 cm^-1^ of native bLTF is the same for AgLTF45, but on the spectrum of AgLTF450, a much more intense band at 1426 cm^−1^ can be observed. Instead, the band at ≈1100 cm^−1^ has not changed its position. On the spectrum of AgLTF45, bands at 1409 and 1381 cm^−1^ have appeared. The respective band may come from the cationic form of the imidazole ring (histidine) [37], confirming its possible interaction with Ag^+^. On the spectrum of AgLTF450, the respective bands may be overlaid by the band at 1426 cm^−1^, and thus cannot be distinguished. The amide III band of native bLTF at 1329cm^−1^ shifted to 1336 cm^−1^ and 1342 cm^−1^ for AgLTF45 and AgLTF450, respectively. Instead, only the intensification of the band at 1280 cm^−1^ was observed. Additional bands assigned to amide III rose to 1246 and 1228 cm^−1^ for AgLTF45 and AgLTF450, respectively. The peak at 1171 cm^−1^ of native bLTF coming from tyrosine shifted to ≈1166 cm^−1^ after Ag^+^ binding. It appeared that the intensity of the phenylalanine bands at 1035, 1003, and 962 cm^−1^ decreased. However, in comparison to the band of C-H stretching at 2931 cm^−1^, no changes were observed. Interestingly, on the spectrum of AgLTF45, the intensity of the S-S bands at 561 and 520 cm^−1^ decreased, with a respective increase in the C-S band stretching at 658 cm^−1^, which may indicate the destruction of disulfide bridges. However, on the spectrum of AgLTF450, the band intensities for both the S-S as well as C-S vibrations increased, indicating that only part of the disulfide bonds may have been ruptured. According to the literature data, Hg^+^ and Ag^+^ are the only metals that can readily split disulfide bridges. Moreover, only Hg^+^ has a higher affinity to sulfhydryl groups than Ag^+^ [9]. Thus, the obtained results are consistent with previously discovered knowledge. 

#### 2.2.4. Fluorescence Study

**Figure 6:**
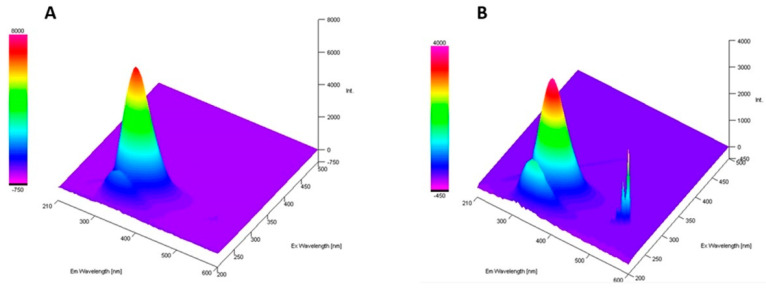
The 3D-spectra fluorescence of (**A**) native bLTF and (**B**) AgLTF1200 complex.

Fluorescence spectroscopy can be utilized to outline the nature of the protein interactions. Native bLTF revealed an emission maximum of tryptophan at λ_em_ = 335 nm, and intensity was nearly 7100 a. u. in case when excitation wavelength of λ_ex_ = 280 was utilized. After Ag^+^ immobilization onto bLTF, the respective emission maximum has not changed, but the fluorescence intensity decreased twofold to ≈3800 a. u. (Figure 6).

Unlike bands of other aromatic residues, the band of the tryptophan emission maximum is highly sensitive to the local environment, which enables it to be utilized for the tracking of changes in protein structure [38]. It was shown that tryptophan embedded deep into a hydrophobic pocket in native folded Azurin has λ_em_ ≈ 308 nm, while fully denatured proteins have λ_em_ ≈ 350 nm [39]. Thus, obtained data showed that no protein unfolding occurred and that the fluorescence quenching has a static character. The charge transfer from ligand to metal should be a reason for such drastic changes, which indicates the Ag^+^ interaction with bLTF through tryptophan residues [40]. However, the additional signal also appeared at λ_ex_/λ_em_ = 270/540 with an intensity of ≈3050 a. u. The respective band should arise due to the formation of AgNPs. N. Paseban et al. investigated AgNPs synthesized with regia green-husk aqueous extract [41]. They showed that as-synthesized NPs depending on excitation wavelength had fluorescence in the range from 487 to 580 nm. The proportional increase in fluorescence from AgNPs to the decrease in tryptophan fluorescence may indicate the transfer of energy adsorbed by tryptophan to newly formed NPs. Still, it noteworthy to mention that tyrosine also adsorbs in the same region [38]. Thus, both of the mentioned residues may be involved in the protein interactions with silver.

#### 2.2.5. Matrix-Assisted Laser Desorption/Ionization Time-of-Flight Mass Spectrometry

**Figure 7:**
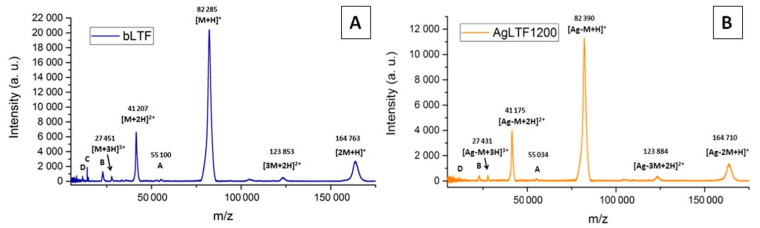
MALDI-TOF MS spectra of (**A**) native bLTF and (**B**) Fe-bLTF complex.

The MALDI-TOF-MS spectrum of native bLTF is shown on Figure 7A. The signal at ≈82.3 kDa represents the protein monomer [M+H]^+^. The signals from the protein dimer [2M+H]^+^ at ≈164.8 kDa and multiple-charged monomer ([M+2H]^2+^ 41.2 and [M+3H]^3+^ 27.4 kDa) or trimer ([3M+2H]^2+^ 123.8 kDa) also can be distinguished on the spectrum. Other signals (from A to D) were assigned to protein impurities, which mainly come from the protein fragmentation during storage or production [22].

After silver immobilization onto bLTF, the decrease in all signals’ intensity can be observed. The signal-intensity decrease is connected to their suppression by unbonded or loosely bonded Ag^+^. Some signals of impurities even become almost undistinguishable. To other effects, the signal shifts to higher *m/z* values can be noticed indicating the formation of silver adducts with protein. Moreover, some signals have shifted to lower *m/z* values, which may be connected to protein oxidation.

#### 2.2.6. SDS-PAGE Analysis

**Figure 8:**
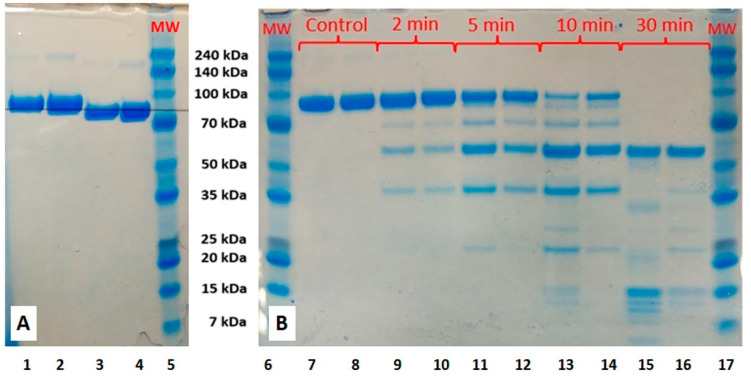
SDS-PAGE of (**A**) bLTF and AgbLTF1200 in reduced (1,2) and nonreduced mode (3,4), and (**B**) the peptic digestion kinetics, where bLTF is presented on 1,3,7,9,11,13,15, AgLTF1200 complex on 1,3,8,10,12,14,16, and markers on 5, 6, 17.

The SDS-PAGE analysis (Figure 8) showed that in both nonreduced and reduced modes, the electrophoretic velocity of AgLTF1200 was slightly higher in comparison to native bLTF. Similar was detected for iron-saturated bLTF in comparison to apo-form. L. Voswinkel et al. investigated the holo-, apo-, and native (partially saturated) forms of bLTF with PAGE-IEF, and they were able to differentiate all of them by comparison of protein-band position [42]. The effect may be connected to the formation of a more compact structure of the protein after Ag^+^ binding. It also explains the differences in protein-band position on the SDS-PAGE performed in reduced and nonreduced modes [43]. However, it is noteworthy to mention that protein reduction with DTT causes the breaking of disulfide bonds and loss of the globular structure. In consequence, no differences in electrophoretic velocity should be observed. Thus, the detected changes may be connected with the influence of the formed AgNPs on the electromigration behavior of synthesized nanocomposite. Interestingly, the study of Zn^2+^ binding to bLTF has revealed the opposite effect, namely that the protein bands appeared slightly higher after metal adsorption [22]. The highest protein-band position was observed for the Zn-LTF complex with the highest Zn^2+^ content. This can be explained by the much lower strength of zinc interaction with bLTF in comparison to silver or iron. Hence, the Zn^2+^ released during the analysis affected the separation process.

#### 2.2.7. Analysis by Capillary Electrophoresis Coupled with ICP-MS

**Figure 9:**
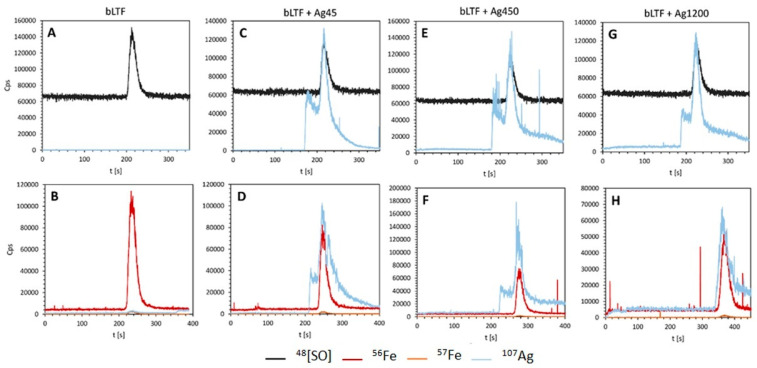
Electropherograms obtained using CE-ICP MS system for native bLTF (**A**,**B**) and AgLTF45 (**C**,**D**), AgLTF450 (**E**,**F**), and AgLTF1200 (**G**,**H**), after applying as a DRC gas oxygen (**A**,**C**,**E**,**G**) or ammonia (**B**,**D**,**F**,**H**).

The capillary electrophoresis (CE) coupled online with ICP-MS was employed to more precisely describe the nature of silver-ion binding by bLTF. Previous research indicated that this approach enables analysis of metal–protein complexes [44]. Figure 9 summarizes the obtained electropherograms for bLTF before and after modification with different initial silver-ion concentrations. 

It was shown that native bLTF does not contain silver. On the other hand, the presence of silver signals on electropherograms for Ag-bLTF complexes confirmed the effective performance of the immobilization process. Moreover, two signals from silver were noted. The first one probably corresponds to unbound or weakly bound silver ions. The second one, occurring at the same retention time as a signal from sulfur oxide (protein) and iron bound with bLTF, can be attributed to silver ions strongly bound with protein. Again, the results are complementary to the isotherm study. The silver in the form of AgNPs is probably strongly bonded to the protein and should correspond to alculated from the Langmuir isotherm. In turn, the loosely bonded silver represents the excess adsorption. The respective silver probably bonded with bLTF through weak electrostatic interactions. However, the free Ag^+^ can also appear as a result of applied current. K. Wonner et al. investigated the oxidative dissolution of single AgNPs during the linear-sweep voltammetry [45]. The dissolution of the AgNPs has already appeared under the current below 1.5 V. Finally, it is noteworthy to mention that the presence of the unchanged signal from iron suggests that the silver ions’ sorption did not affect ability of the protein to bind to iron. Moreover, as it was shown previously, even the highest Ag^+^ concentration did not cause significant Fe^3+^ elimination from bLTF (the reaction was performed in acidic conditions pH = 5). Instead, when bLTF was incubated with Zn^2+^, the loss in Fe^3+^—depending on the utilized conditions—could reach 45% [22].

#### 2.2.8. Ag^+^ Desorption Study

Silver desorption was performed in three different buffers that imitate conditions in different parts of the digestive tract. The choice of the utilized buffers was performed taking into account the “Dissolution test for solid dosage form” standardized by European pharmacopoeia (EP). However, normally in order to provide appropriate conditions, the 0.1 M hydrochloric solution and phosphate buffers are utilized. Unfortunately, chloride and phosphate ions form insoluble salts with silver. Thus, we selected the MOPS buffer for pH 6.8 based on L. Babel et al.’s work [46], acetate buffer pH 4.5 recommended by EP as alternative to phosphate, and HNO_3_ solution pH 1.2. After 2 hours of the desorption process, the released silver was quantified by ICP-MS. The desorption was calculated as 80.5, 64.3, and 72.2 % at pH 1.2, 4.5, and 6.8, respectively, with actual silver concentrations in the filtrate of 1.89, 1.51, and 1.70 ppm. One of the mechanisms of silver NPs and nanocomposites’ cytotoxicity is attributed to the possibility of Ag^+^ release. It is extensively discussed in literature and acquired the name “Trojan horse” mechanism [4]. Thus, numerous studies have been performed to evaluate the influence of different conditions on the Ag^+^ release. Among the factors that have the impact on the process are the presence of oxygen, light irradiation, temperature, and pH. For instance, Kosa et al.’s work shows that at basic pH 10.0 the dissolution was almost absent (0.8 × 10^−4^ s^−1^), while at neutral and acidic pH of 7.0 and 4.0 it was fast and differed only on a few tenths of a unit (2.5 and 1.8 s^−1^, respectively) [47]. The presence of the different ligands in the solution should also have an impact on the dissolution rate, which explains the higher desorption at pH 6.8 in comparison to pH 4.5. Taking into account the assumption of the mentioned theory, the high Ag^+^ desorption rate from the obtained complex could raise anxiety about safety of the synthesized complexes. However, according to the work presented in Hadrup et al., the orally administrated silver in 90–98% undergoes fecal excretion depending on the species. [48]. Still, it was shown that negative toxic effects can be observed for cells exposed to Ag^+^ concentration of even 0.5 ppm [49].

### 2.3. The Study of Complex Biological Activity

#### 2.3.1. Assessment of the Impact of Ag^+^ Binding to bLTF on Its Susceptibility to Peptic Degradation

To complement the Ag^+^ desorption study, an assessment of the AgbLTF1200 complex’s susceptibility to pepsin digestion was performed. The peptides, compared to proteins, are more effectively absorbed in the gastrointestinal tract [50]. Thus, the efficiency of nanocomposite digestion should provide additional information about its safety. Figure 8B shows the SDS-PAGE analysis that represents the kinetic study of bLTF peptic digestion. After 5 and 10 min of digestion, for native bLTF compared to AgLTF1200 the appearance of a higher amount of peptides in a mass range between 35 and 70 kDa can be observed. Instead, the band corresponding to intact protein decreased slightly faster in the case of native bLTF. The results may be connected with the Ag^+^ release from the nanocomposite. Previous studies showed that Ag^+^ released from nanocomposites, with its subsequent binding to proteins, in particular enzymes, disturbs their function [4]. Thus, it can be attributed to one of the possible mechanisms of silver nanocomposites’ cytotoxic and antibacterial properties. However, our study showed that changes in the peptic digestion process are much more complex than it may seem on first sight. In case, when protein was subjected for digestion during 10 and 30 min, the decrease in bigger peptides occurred much faster in the case of AgbLTF1200. This fact may indicate that Ag^+^ binding to bLTF induces changes in the protein structure, which makes the cleavage of places more accessible for enzymes [51]. In general, the Ag^+^ binding to protein almost did not influence the pepsin activity, while changes in structure even sped up the digestion process. 

#### 2.3.2. Determination of Minimum Inhibitory Concentration

Diabetic foot infection (DFI) is the most common compilation of diabetes, which is strictly connected with the formation of chronic wounds. Lately, DFI has become an issue of a global range and has magnified significantly as the population of diabetes has increased. Thus, we decided to evaluate the antibacterial properties of the synthesized complex against the three most common bacterial strains isolated from DFI wounds [52]: two Gram-positive *S. aureus* and *E. faecalis,* and one Gram-negative *P. aeruginosa*. Moreover, it was shown that topical application of lactic-acid bacteria (LAB) can improve wound healing, which encouraged us to examine the toxic effect of AgbLTF1200 onto two of them, namely *L. lactis* and *L. paracasei* isolated from fermented vegetables. 

**Figure 10:**
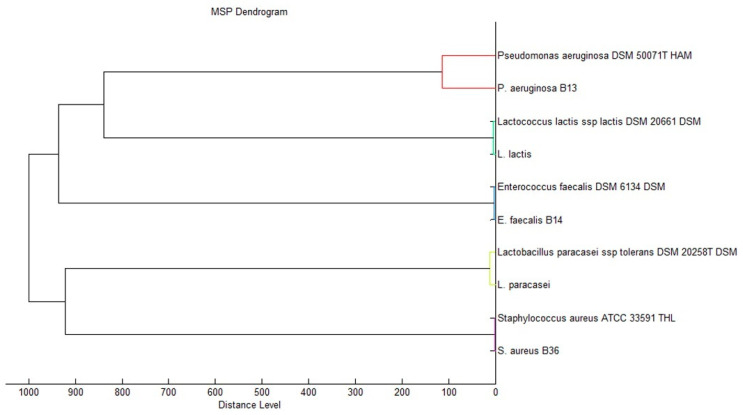
Phyloproteomic tree of the isolated bacterial strains, identified by MALDI Biotyper Compass platform.

All of the as-mentioned bacteria were identified with utilization of the MALDI-TOF-MS technique based on the Raw spectra. The identification was performed according to the standard protocol supplied by the equipment and software producer (Bruker Daltonics, Bremen, Germany), i.e., by the analysis of bacterial-protein extracts obtained with the use of 70% formic acid. The bacteria were identified with a high confidence level, Score Value ≥1.999, i.e., *S. aureus* 2.380; *E. faecalis* 2.420; *P. aeruginosa* 2.160; *L. lactis* 2.280; *L. paracasei* 2.220. Figure 10 shows the phyloproteomic relationships of the identified bacteria. The most closely related strains identified by MALDI Biotyper Compass platform are also included. 

Despite multiple reports about antibacterial properties of lactoferrins [15,17,19,20], in our investigations the studied bacteria did not show susceptibility to bLTF with concentration up to 5 mg/mL. In previous studies of our group, bLTF also did not show antibacterial activity against *S. aureus* and *E. faecalis* from ATCC collection, but slight inhibition of *P. aeruginosa* ATCC 27853 was revealed [10]. Still, only 75.7% inhibition of *P. aeruginosa* ATCC 27853 growth was observed when bLTF in concentration of 50 µg/mL was utilized. The lower antibacterial activity of bLTF observed in the present study may be connected with its iron saturation, namely the holo-bLTF that was utilized. Instead, the saturation level was not examined in 2016. In turn, for all pathogenic bacteria, an MIC value of Ag^+^ was established at 80 µg/mL, while for LAB it was 40 µg/mL. The combination of silver with bLTF significantly increased the antibacterial activity of the AgbLTF1200 in comparison to both bLTF and Ag^+^. The MIC value of AgbLTF1200 for *S. aureus* and *E. faecalis* was determined as 1.25 mg/L and 0.625 mg/L for the rest of the tested bacteria, which was 5.0 and 2.5 µg/L of Ag^+^, respectively. The lower MIC values could be due to the formation of AgNPs during the reaction of Ag^+^ with bLTF. It was shown that AgNPs revealed higher toxicity against both eucaryotic and procaryotic cells [4]. Moreover, bLTF can promote Ag^+^’s entrance inside the bacterial cells, thus showing the synergistic effect of antibacterial action. In P. Pomastowski et al.’s work, the antibacterial properties of the synthesized Ag-bLTF complex were much higher. The growth of some bacteria was completely inhibited with the Ag-bLTF concentration of 0.05 mg/mL. This could be connected to the preparation procedure, which did not include the washing steps; thus, a higher amount of silver should remain in the complex [10]. Interestingly, the combination of bLTF with other d-metals can lead to the formation of complexes that promote the growth of the LAB [53]. It was reported previously that bLTF itself can promote LAB growth [19]. However, K. Śpiewak et al. showed in their work that only high bLTF concentration in both apo- and holo-form (e.g., 40 mg/mL) slightly influenced the growth of *L. plantarum*. Instead, Mn_2_-bLTF even in concentration of 0.6 mg/mL promoted their growth. 

#### 2.3.3. Cytotoxicity Study

**Figure 11:**
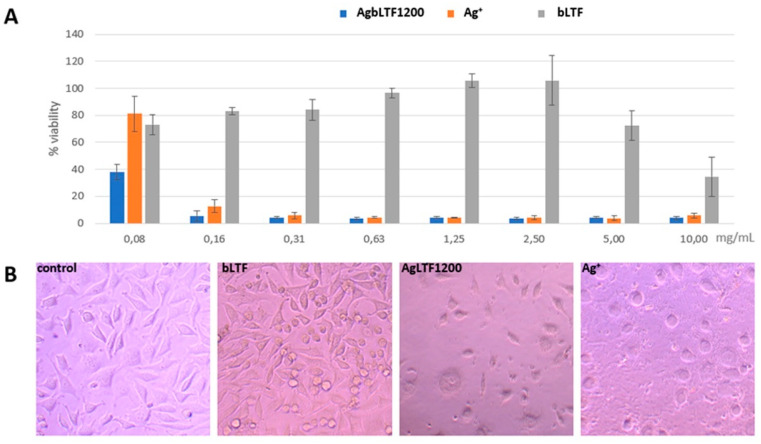
(**A**) Represents cell viability measured by MTT assay. Histograms show the percentage, with respect to control cells (100%), of viable cells after exposure to AgLTF1200, Ag^+^ and bLTF. Data show the mean ± SE (n = 5); (**B**) Morphology of L929 control cells and after exposure to bLTF, AgLTF1200, and Ag^+^.

Cytotoxicity of AgbLTF1200 was determined by the MTT method on the L929 mouse fibroblast cell line. This cell line is usually used for testing the biocompatibility of new materials according to ISO 10993-5 and ISO 10993-12 norm. In our experiment, both AgbLTF1200 and Ag^+^ were utilized. The concentration of the complex was in the range from 0.08 mg to 10 mg/mL, which corresponds to the Ag^+^ concentration from 0.32 to 40 µg/mL, respectively. The results are presented in Figure 11. 

When a complex with a concentration above 0.16 mg/mL (Ag^+^ 0.64 µg/mL) was utilized, the cell viability significantly decreased to below 20% (Figure 11A). The same was observed for the Ag^+^ solution of respective concentration. At a complex concentration of 0.08 mg/mL, cell viability for AgbLTF1200 was about 40%, whereas for the corresponding Ag^+^, concentration was two times higher. This may suggest that bLTF and silver act synergistically and that lactoferrin may promote Ag^+^ entry into cells. The results are complementary to the results of antimicrobial activity, where AgLTF1200 had much lower MIC in comparison to Ag^+^ silver. Pure bLTF did not display cytotoxicity in the range from 0.08 to 5 mg/mL. Only the highest tested concentration, i.e., 10 mg/mL, caused a decrease in cell viability to about 40%. In the sample treated with AgbLTF1200 (10 mg/mL), there were significantly fewer cells compared to the control, but most of them still adhered to the substrate (Figure 11B). Only single ones changed the shape to the rounded. In samples treated with Ag^+^, all cells showed a balloon-like cell shape and expanding cytosol, indicating the activation of the apoptosis process. Evidently, the AgLTF1200 complex has a different mechanism of action, which may change the processes that take part in cell division, but does not activate the apoptosis. 

## 3. Materials and Methods

### 3.1. Chemicals and Materials

Sigma-Aldrich (Steinheim, Germany) supplied the following chemicals and materials: lactoferrin from bovine milk (bLTF), ammonium iron (III) citrate, sodium chloride, sodium hydroxide, MS-grade nitric acid, hydrochloric acid, Amicon^®^ Ultra Centrifugal membrane filters, ICP multi-element standard solution IV and scandium standard solution for ICP, trypsin, fetal bovine serum (FBS), Dulbecco’s Modified Eagle’s Medium (DMEM), phosphate-buffered saline (PBS), EDTA, penicillin-streptomycin solution, L929 normal mouse fibroblast cells and Caco-2 cells, MTT assay kit, and LDH release assay kit. Invitrogen Bolt^TM^ 4–12% Bis-Tris Plus polyacrylamide gel 12 wells, Simply Blue^TM^ Safe Stain (Coomassie G_250_ stain), 20X MES SDS Running Buffer, load sample buffer, sample reducing agent, and borosilicate microscope slides were obtained from ThermoFisher Scientific (Waltham, MA, USA). Perfect^TM^ Color Protein Ladder was from EUR_X_ Sp. z o.o. (Gdansk, Poland). Set of automatic pipettes and laboratory plastics were obtained from Eppendorf (Hamburg, Germany). Moreover, deionized water was obtained with Milli-Q RG system from Millipore (Millipore Intertech, Bedford, MA, USA).

### 3.2. Batch-Sorption Analysis

#### 3.2.1. Preparation of Solutions for the Batch-Sorption Study and Complex Synthesis

All Ag^+^ solutions were prepared in deionized water with no additional modifications. The exact Ag^+^ concentration in the solutions was quantified by ICP-MS analysis. Here and in all other analyses, metal quantification was performed on Shimadzu ICP-MS 2030 (Kyoto, Japan) with scandium (^45^Sc) as an internal standard. The quantification was performed taking into account the ^107^Ag isotope. The 1% HNO_3_ was utilized for the dilution. Instead, the bLTF suspension was prepared in deionized water with concentration of 5 mg/mL (by weight) and adjusted to pH 5.0. The pH adjustment was performed with 1% HNO_3_ or 0.1 NaOH. For the study, bLTF standard purchased from Sigma-Aldrich (Steinheim, Germany) was utilized. Before the study, the physicochemical characterization of the bLTF was performed; among others, the iron content was determined. According to obtained data, the utilized bLTF was in holo-form. The results are presented in the article by O. Pryshchepa et al. (2022) [22]. The bLTF from the same batch was utilized in both studies. 

#### 3.2.2. Isotherm Study of Ag^+^ Adsorption

Study was performed with utilization of Ag^+^ solutions with concentration of 6, 15, 30, 45, 60, 120, 300, 450, 600, 900, and 1200 mg/L. bLTF suspension was mixed with Ag^+^ solution (1:1 ratio) in black polypropylene microcentrifuge tubes. The samples were incubated for 24 h at room temperature (≈23 °C). Subsequently, the mixtures were filtrated through Amicon^®^ Ultra Centrifugal Filters cut-off 3 kDa at room temperature. The parameters for centrifugation were as follows: room temperature, 20,000× *g*, 5 min. The filtrate was subjected to quantification of Ag^+^, which remained in the solution by ICP-MS. Instead, the retentate containing the synthesized AgLTF complexes was washed twice with deionized water, collected to Eppendorf tubes, and lyophilized for subsequent analyses. The respective complexes were encoded as AgLTF6-AgLTF1200 depending on the amount of silver that was utilized for the synthesis. 

The Ag^+^ adsorption onto protein was calculated accordingly:

(1) $$q_{e}=\frac{\left(C_{0}-C_{e}\right)\times V}{m}$$

where *q_e_* is the Ag^+^ adsorption on protein, *m* is the mass of the bLTF in the reaction mixture (g), *C*_0_ and *C_e_* are the initial and equilibrium concentration of Ag^+^ in the reaction mixture (mg/L), and *V* is the volume of the reaction mixture (L). 

The sorption efficiency (*E*) was calculated and expressed in % according to:

(2) $$E=\frac{100\cdot \left(C_{0}-C_{e}\right)}{C_{0}}$$

The changes in the Gibbs free energy were calculated as follows [10,24]:

Δ*G* = −*RT lnK_D_* (3)

where Δ*G* is the free energy change in kJ/mol (the adsorption energy), *R* is the gas constant (8.314 J/mol·K), *T* is the reaction absolute temperature in Kelvin (295 K), and *K_D_* is the distribution coefficient. 

The K_D_ coefficient expresses the affinity of metal to the protein and can be calculated according to the equation:

*K_D_* = *q_e_*/*C_e_* (4)

where *q_e_* is the amount of Ag^+^ adsorbed onto bLTF (mg/g) and *C_e_* is the equilibrium concentration of Ag^+^ in solution (mg/L). 

The modeling of the adsorption data was performed with utilization of Freundlich and Langmuir isotherms and isotherm as function of *C_e_/C*_0_ [11].

### 3.3. Physicochemical Characterization of Synthesized Complexes

#### 3.3.1. Electron Microscopy Studies

The investigations were conducted with scanning electron microscope (SEM) capable of imaging in scanning transmission electron microscopy (STEM) mode, model Hitachi S5500 equipped with EDX system. Observations were performed under accelerating voltage of 30.0 kV in transmission and surface mode. Samples were prepared by dispersion in deionized water. Droplets of the solution were deposited onto carbon-coated copper TEM grids (Lacey Carbon Support Film 400 mesh; Electron Microscopy Sciences) and dried on air at room temperature. STEM and SEM observations were performed using secondary electron (SE) detector and STEM detector. X-ray mode in DF (dark field) phase contrast and BF (bright field) contrast were used. Moreover, as-prepared samples of AgLTF45, AgLTF450, and AgLTF1200 were subjected to TEM-SAED. The analysis was performed on FEI Tecnai F20 X-Twin (Hillsboro, OR, USA).

#### 3.3.2. Fourier Transform Infrared Spectroscopy (FTIR)

FTIR analysis of the synthesized complexes and native bLTF was carried out in the MIR range (4000–400 cm^−1^) with utilization of attenuated total reflection (ATR) mode on Alpha FTIR spectrometer (Bruker, Billerica, MA, USA). Normalized FTIR spectra were plotted using the Origin software (v. 2015, OriginLab Corporation, Northampton, MA, USA) followed by normalization operation. 

#### 3.3.3. RAMAN Spectroscopy 

The RAMAN spectra were recorded on SENTERRA II Dispersive Raman Microscope (Bruker, Billerica, MA, USA) in the MIR range and excitation wavelength at λ = 532 nm. Raman spectra were processed (normalization/baseline correction) and plotted using the Origin software (v. 2015, OriginLab Corporation, Northampton, MA, USA).

#### 3.3.4. Fluorescence-Spectroscopy Analysis

The solutions of bLTF and AgLTF1200 complex with concentration of 0.25 mg/mL were prepared in deionized water. The 3-D fluorescence spectra were obtained with utilization of Jasco FP-8300 spectrofluorometer (JASCO, Easton, MD, USA) in the excitation range λ = 200–735 nm and emission range λ = 210–750 nm.

#### 3.3.5. Matrix-Assisted Laser Desorption/Ionization—Time-of-Flight Mass Spectrometry (MALDI-TOF-MS) 

Sinapinic acid (SA) was utilized as a matrix for MALDI analysis. Intact protein and AgLTF1200 complex were suspended in 0.1% trifluoroacetic acid to concentration of ≈1 mg/mL. The saturated solution of SA was prepared in the TA30 solvent (30:70 [*v*/*v*] acetonitrile: 0.1% trifluoroacetic acid). A total of 1 µL of the samples were applied on GroundSteeltarget plate by dried-droplet technique and overlaid with 1 µL of the matrix solution. Protein-calibration standard II was used for mass calibration. All the MS spectra were obtained using the MALDI-TOF/TOF mass spectrometer UltrafleXtreme (Bruker Daltonics, Bremen, Germany) equipped with modified neodymium-doped yttrium aluminum garnet (Nd:YAG) laser operating at the wavelength of 355 nm and frequency of 2 kHz. The system was controlled using the Bruker Daltonics software (flexControl and flexAnalysis). MS spectra were obtained in the linear positive mode in an *m/z* range of 5000−200,000, applying an acceleration voltage of 25 kV. Spectra were acquired by summing up three individual spectra obtained with 500 laser shots each and were plotted using the Origin software (v. 2015, OriginLab Corporation, Northampton, MA, USA) from raw data without any modifications. 

#### 3.3.6. Ag^+^ Desorption Study

Silver desorption tests for AgLTF1200 were performed in three buffers, namely in HNO_3_ solution pH 1.2, acetic buffer pH 4.5 [54] and 0.1 M MOPS buffer pH 6.8, which imitates acid-base conditions in different parts of the digestive tract. The AgLTF1200 suspension with concentration of 10 mg/mL was prepared in deionized water. Next, 50 µL of the suspension (≈0.5 mg of the complex) was transferred to the Eppendorf tubes and mixed with 200 µL of buffers. The mixtures were incubated for 2 h at 37 °C. Subsequently, mixtures were filtrated with Amicon^®^ Ultra Centrifugal Filters cut-off 3 kDa. Finally, the Ag^+^ content in the filtrate was determined. 

At the same time, the silver and iron content in the complex was determined. For the complex mineralization, 50 µL of suspension (≈0.5 mg of AgLTF1200) was transferred to Eppendorf tubes and mixed with 0.2 mL of MS-Grade nitric acid. Next, tubes were closed tightly and heated at 80 °C for 3 h. Then, the obtained samples were diluted with deionized water and subjected to ICP-MS analysis.

#### 3.3.7. SDS-PAGE Analysis

The samples with concentrations of ≈0.5 mg/mL were prepared in deionized water. Next, the protein solutions were analyzed with utilization of 4–12% Bis-Tris Plus polyacrylamide gel (Thermo Scientific, Waltham, MA, USA) in reduced and nonreduced mode, and 1X MES Running Buffer (0.05M MES, 0.05 M Tris, 1 mM EDTA and 0.1% SDS, pH—7.3) was applied according to the standard procedure recommended by manufacturer. Gel staining was performed with Coomassie Blue R-350 ready-to-use stain. 

#### 3.3.8. Analysis by Capillary Electrophoresis Coupled with ICP-MS

For the capillary electrophoresis separation, the lyophilized powder of protein and complex were suspended separately in borate buffer (30.183 g of Na-Borat in 0.5 L of water; pH = 7.8) to final concentration of 1 mg/mL. The separation was performed in noncoated fused silica capillary (90 cm × 75 μm ID) with usage of PrinCe 760 capillary electrophoresis (CE) system (Prince Technologies B.V.). Each analysis was preceded by rinsing the capillary sequentially for 1 min with: 0.1 M NaOH, Milli-Q water and borate running buffer. The applied pressure during the sample injection was 250 mbar for 20 s. The CE separation was performed using +20 kV voltage and 250 mbar pressure. 

The CE-capillary was connected to the NexIon 300D ICP-mass spectrometer (Perkin Elmer) using an inhouse interface [55]. This setup enabled element-selective electropherograms to be directly monitored on-line by ICP-MS using dynamic reaction cell (DRC) mode at isotopes ^48^[SO], ^56^Fe, ^57^Fe, and ^107^Ag. For each sample, analysis was performed using two types of DRC gas. For detection of iron isotopes, the DRC gas was ammonia (0.6 mL/min, RPq = 0.45) and for detection of sulfur as ^48^[SO] oxygen was used. Both DRC gases allowed detection of silver isotopes. The RF power was 1250 W and plasma gas was set at 16 L Ar/min. Nebulizer gas flow rate was established at 0.98 Ar/min. 

### 3.4. The Study of Complex Biological Activity

The assessment of biological activity was performed for AgLTF complex synthesized with utilization of Ag^+^ solution of 1200 mg/L (AgbLTF1200). 

#### 3.4.1. Evaluation of the Impact of Ag^+^ Binding to bLTF on Its Susceptibility to Peptic Degradation

The analysis was performed in simulated gastric fluid free of pepsin according to adjusted protocol from R. Wang et al. [50] The media was prepared as follows: 2.0 g of sodium chloride was mixed with 80 mL of 1 M hydrochloric acid and diluted to 1000 mL. The pepsin stock solution with the concentration of 2000 U/mL was prepared in deionized water considering the enzyme activity declared by supplier in certificate of analysis. Subsequent enzyme dilutions were performed with simulated gastric fluid to obtain the solution with concentration of 5 U/mL. The native bLTF and Ag-bLTF complex was suspended in deionized water to concentration of 5 mg/mL. Next, the mixture with enzyme-to-substrate ratio of 0.1 U:100 µg was prepared in reaction media, where the protein’s final concentration was 1 mg/mL. The mixture was incubated at 37 ℃ for 2, 5, 10, and 30 min. The reaction was terminated by addition of 0.7 M Na_2_CO_3_ at 35% of the reaction volume, namely 35 µL of solution being added to 100 µL of reaction mixture. The control samples were treated in the same manner with the incubation time of 30 min, but with the addition of appropriate amount of buffer instead of pepsin solution. Next, all prepared samples were diluted twice and subjected to SDS-PAGE analysis in reduced mode.

#### 3.4.2. Determination of Minimum Inhibitory Concentration (MIC)

MIC was determined by broth microdilution method with the use of 96-well plates and Mueller–Hinton broth (MHB) as a culture medium. The antibacterial agent solutions were prepared in MHB by serial dilution method. The Ag^+^ solutions with concentration of 1.25, 2.5, 5.0, 10.0, 20.0, 40.0, 80.0, and 160 µg/mL were tested. For bLTF and AgLTF1200, the solutions with concentration of ≈ 0.31, 0.62, 1.25, 2.5, 5.0, and 10.0 mg/mL were prepared. The MIC determination was on the microorganisms from deposit of Centre for Modern Interdisciplinary Technologies Nicolaus Copernicus University in Torun, performed for three pathogenic bacterial strains isolated from diabetic foot wounds *S. aureginosa, S. aureus, and E. fecalis* (permission of Ethic Committee of NCU in Toruń KB 68/20019) and two probiotic bacteria isolated from fermented vegetables *L. lactis* and *L. paracasei*. A 24 h bacterial culture was diluted to the concentration of 0.5 McFarland, which was further diluted 100-fold. The prepared bacterial suspension was mixed with antibacterial agent solutions at a ratio of 1:1. The negative control and positive control were also performed. The incubation was carried out at 37 ℃ for 24 h with mixing. The detection was performed based on the fluorescence measurements with utilization of microplate reader (Multiskan, ThermoFisher) and in vitro Toxicology Assay Kit, resazurin-based (Sigma-Aldrich) according to protocol provided by kit supplier. 

#### 3.4.3. Cytotoxicity Study

L929 cell line of mouse fibroblasts from European Collection of Authenticated Cell Cultures operated by Public Health England (Sigma) were cultured in DMEM supplemented with 10% (*v/v*) fetal bovine serum, 2 mM glutamine, and 100 U/mL penicillin, and 100 μg/mL streptomycin (Sigma). These cell lines were cultured in 75 cm^2^ flasks at 37 °C and 5% CO^2^. The cells were harvested by trypsinization using 0.25% trypsin/EDTA every 3–4 days. 

For MTT assays, cells were cultured on 96-well plates at density 2 × 10^5^ cells/mL and incubated for 24 h. Then, the medium was replaced with a new one containing Ag^+^ of the appropriate concentration and incubated for 24 h. Then, 10% (*v/v*) of Thiazolyl Blue Tetrazolium Bromide (MTT) solution (5 mg/mL in PBS) was added and incubated for 4 hours at 37 °C. Next, the medium from wells was removed and the formazan crystals were dissolved in DMSO for 10 min by mixing. Absorbance was measured using a microplate reader (Multiskan, ThermoFisher) at 570 nm and 650 nm as background absorbance.

## 4. Conclusions

In conclusion, we have investigated the sorption process of Ag^+^ onto bLTF based on the batch isotherm study. The results indicate that the approach can be utilized for the prediction of the metal–protein complexes’ features. Based on isotherm results, selected AgbLTF complexes were subjected to multi-instrumental analysis. The electron microscopy revealed the formation of nanoparticles. However, the capillary electrophoresis results also indicate that part of the Ag^+^ should remain in ionic form and bond with protein through weak interaction forces. The spectroscopic techniques enabled us to predict places of silver binding to protein, e.g., amino acids such as tryptophan or methionine, but also nitrogen of peptide bonds and other heteroatoms. Finally, the biological activity of AgbLTF1200 was estimated. The results revealed that the combination of bLTF with silver has a synergistic effect. Thus, the obtained complex has higher toxicity against both the procaryotic and eucaryotic cells. Moreover, the changes in morphology of L929 mouse fibroblast indicates that the mechanism of its biological activity differs from the toxic mechanisms of Ag^+^ itself, which requires additional studies. Still, the obtained complex has a promising application as an antibacterial agent in the treatment of chronic wounds; among others, the wounds accompanying the diabetic foot infections. However, it requires extended studies with utilization of animal models, e.g., murine or porcine skin models, to evaluate the possible benefits and risks of the obtained product.

## Figures and Tables

**Table 1:** Calculated values derived from Freundlich and Langmuir isotherm models.

Freundlich Isotherm				Langmuir Isotherm			
K_F_ [mg/g]	1/n	S	R^2^	K_L_ [L/mg]	*q_m_* [mg/g]	S	R^2^
8.465	0.213	3.45	0.905	19.94	1.55	1.93	0.971

## Data Availability

Not applicable.
